# 

**Published:** 1896-02

**Authors:** 


### CONTENTS.

ORIGINAL ARTICLES:
- The Technique of the Tuberculin Test. By G. A. JOHNSON, D.V.M. . . . . 85
- Undescended Testicle (in the Horse). By S. SISSON, V.S. . . . . . . 94
- Report of the Chairman of the Committee on Intelligence and Education . . 97
- Pyæmia in the Dog. By HARRI H. DELL . . . . . . . . . . 100
- Some Diseases of the Hock-joint. By W. J. MARTIN, V.S. . . . . . . 104
- Trichinosis. By OTTO NOACK, V.S. . . . . . . . . . . . 108
- Actinomycosis. By JOHN LATHAM . . . . . . . . . . . 116

Abstracts from Foreign Journals . . . . . . . . . . . . . 120

REPORTS OF CASES:
- Peculiar Fatal Result of an Œsophageal Obstruction. By E. B. THURSTON . 130
- Golden-rod Killing Horses. By J. L. SCOTT, V.S. . . . . . . . 132

EDITORIALS:
- Better Remuneration for Scientific Skill Recognized—Advertising One's Profession and Proprietary Remedies—Legislation in Iowa—McKillip's Loss—An Unfortunate Incident . . . . . . . . . . . . . . 135–137

LEGISLATION—New York State Veterinary Legislation—Iowa . . . . 138–143

CONTROL WORK . . . . . . . . . . . . . . . . . 144

SOCIETY PROCEEDINGS:
- Montreal Veterinary Medical Association—California State Veterinary Association—Manitoba Veterinary Medical Association—Missouri Valley Veterinary Association—Ontario Veterinary Association, Toronto, Canada—Michigan State Veterinary Association—District of Columbia Veterinary Medical Association—Keystone Veterinary Medical Association—New York County Veterinary Medical Association . . . . . . . . . . . . . 152–162

CHIT-CHAT . . . . . . . . . . . . . . . . . . 163

PERSONAL . . . . . . . . . . . . . . . . . . 166

NEW INVENTIONS . . . . . . . . . . . . . . . . 168

